# *Wolbachia* in mosquitoes from the Central Valley of California, USA

**DOI:** 10.1186/s13071-020-04429-z

**Published:** 2020-11-10

**Authors:** Ryan Torres, Eunis Hernandez, Valeria Flores, Jose Luis Ramirez, Andrea L. Joyce

**Affiliations:** 1 Public Health, University of California, 5200 North Lake Road, Merced, CA 95343 USA; 2 USDA-ARS, NCAUR, Crop Protection Research, 1815 N. University, Peoria, IL 61604 USA

**Keywords:** *Wolbachia*, Strain characterization, Supergroup, *16S* rRNA, Multilocus sequence typing (MLST), *Culex pipiens*, *Culex stigmatosoma*, *Culiseta inornata*, *Aedes melanimon*, *Aedes aegypti*, Vector control

## Abstract

**Background:**

*Wolbachia* bacteria are widely distributed throughout terrestrial arthropod species. These bacteria can manipulate reproduction and influence the vector competence of their hosts. Recently, *Wolbachia* have been integrated into vector control programmes for mosquito management. A number of supergroups and strains exist for *Wolbachia*, and they have yet to be characterized for many mosquito species. In this study, we examined *Wolbachia* prevalence and their phylogenetic relationship to other *Wolbachia*, using mosquitoes collected in Merced County in the Central Valley of California.

**Methods:**

Adult mosquitoes were collected from 85 sites in Merced County, California in 2017 and 2018. Traditional and quantitative PCR were used to investigate the presence or absence and the density of *Wolbachia*, using *Wolbachia*-specific *16S* rRNA and *Wolbachia-*surface protein (*wsp*) genes. The supergroup of *Wolbachia* was determined, and Multilocus Sequence Typing (MLST) by sequencing five housekeeping genes (*cox*A, *gat*B, *fts*Z, *hcp*A and *fbp*A) was also used to determine *Wolbachia* supergroup as well as strain.

**Results:**

Over 7100 mosquitoes of 12 species were collected: *Aedes* *melanimon*, *Ae*. *nigromaculis*, *Ae*.* vexans*, *Ae*.* aegypti*, *Culex pipiens*, *Cx*.* stigmatosoma*, *Cx*.* tarsalis*, *Anopheles franciscanus*, *An*.* freeborni*, *An*.* punctipennis*, *Culiseta incidens* and *Cs*.* inornata*. Eight showed evidence of *Wolbachia*. To our knowledge, this study is the first to report detection of *Wolbachia* in five of these species (*Ae*.* melanimon*, *Cx*.* stigmatosoma*, *Cx*.* tarsalis*, *Cs*.* incidens* and *Cs*.* inornata*). *Culex pipiens* and *Cx*.* stigmatosoma* had a high frequency and density of *Wolbachia* infection, which grouped into supergroup B; *Cs*.* inornata* clustered with supergroup A. MLST comparisons identified *Cx*.* pipiens* and *Cx*.* stigmatosoma* as *w*Pip strain type 9 supergroup B. Six species had moderate to low (< 14%) frequencies of *Wolbachia*. Four species were negative, *Ae*.* nigromaculis*, *An*.* franciscanus*, *An*.* freeborni* and *Ae*.* aegypti*.

**Conclusions:**

New records of *Wolbachia* detection were found in mosquitoes from Merced County, California. *Culex stigmatosoma* and *Cs*.* inornata* were new records for *Wolbachia* supergroup B and A, respectively. Other species with *Wolbachia* occurred with low frequency and low density. Detection of *Wolbachia* in mosquitoes can be used to inform potential vector control applications. Future study of *Wolbachia* within *Cx*.* stigmatosoma* and *Cs*.* inornata* in California and through the range of these species could further explore *Wolbachia* infection in these two species.
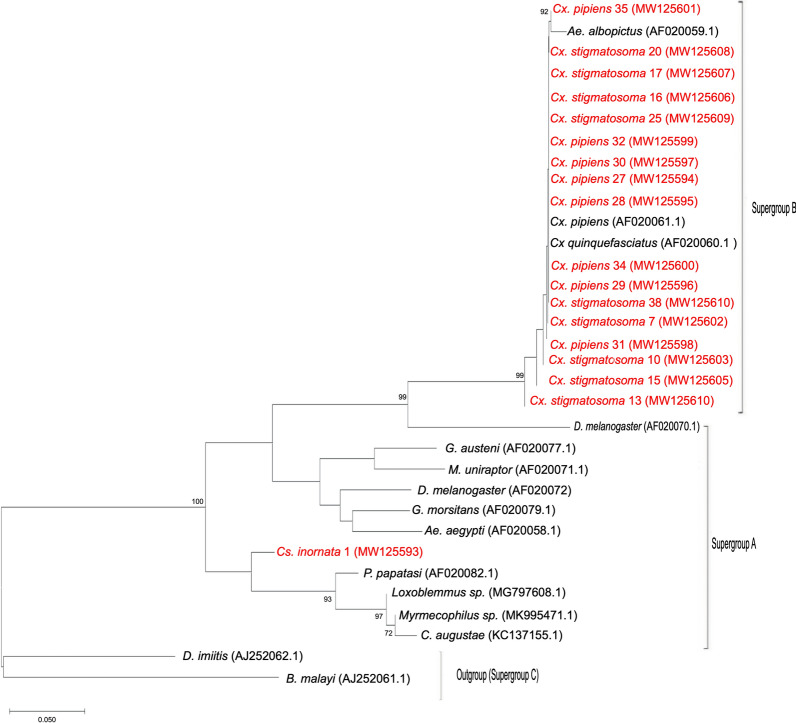

## Background

*Wolbachia pipientis* are a monophyletic group of obligate intracellular bacteria that belong to the order *Rickettsiales*. These endosymbionts were first discovered in the *Culex pipiens* mosquito [1, 2], and are now estimated to infect between 40–52% of arthropod species [3, 4]. *Wolbachia* routinely infect their host’s reproductive tissues, and they are capable of surviving in a variety of invertebrates [5–7]. *Wolbachia* are known to be transmitted vertically through maternal inheritance and have also been shown to transmit horizontally between species, genera, and orders [8–11]. *Wolbachia* infections can have a diverse range of effects depending on the host species, from commensal, mutualistic, to parasitic interactions [5].

In recent years, *Wolbachia* have been implemented for population control of vector species [12, 13]. This is largely a result of the reproductive alterations that *Wolbachia* induce within their hosts in a strain-specific manner [5, 14]. Such reproductive manipulations include termination of male offspring, feminization of genetic males, parthenogenesis, and cytoplasmic incompatibility [5, 15]. Cytoplasmic incompatibility is the only known phenotype to be expressed within mosquito species [16]; when infected males mate with uninfected females, viable offspring are not produced. Furthermore, *Wolbachia* have been shown to modulate host fitness and vector potential. For instance, studies have shown a protective effect of *Wolbachia* against infection with pathogenic RNA viruses [12, 14, 17–21]. In addition, *Wolbachia-*infections have shown other complex host-specific manipulations: they can have increased or decreased rates of reproductive phenotypes; reduced host life-span and egg viability [6, 22]; impact larval survival [23]; decreased female mosquito biting ability [24]; decreased relative abundance of resident bacteria [25]; and in some cases, increased viral susceptibility and host mortality [26, 27]. Artificial infection of this endosymbiont into arthropod vectors has been shown to impact transmission of vector-borne diseases including lymphatic filariasis, West Nile virus, chikungunya, dengue, Zika, and avian malaria [14, 19, 20, 26].

*Wolbachia* offers a potential effective alternative to traditional chemical pesticide applications for the control of disease vectors, for example through cytoplasmic incompatibility. Cytoplasmic incompatibility was first proposed as a method of biological control for *Culex pipiens fatigans* in 1967, although initially it was not attributed to *Wolbachia* [28]. Since then, the use of *Wolbachia*-mediated incompatible technique strategies have been studied for pest control of a number of insects including *Aedes aegypti*, *Ae*.* albopictus*, *Ae*.* polynesiensis*, *Ceratitits capitata*, *Rhagoletis cerasi*, *Glossina morsitans*, *Culex pipiens* and *Cx*.* quinquefasciatus* [28–34]. This method of control aims to reduce vector populations through the introduction of *Wolbachia*-infected ‘sterile’ males, which compete with uninfected males for mates at the release site. *Aedes aegypti* do not naturally harbor *Wolbachia*; when *Wolbachia* have been detected in *Ae*.* aegypti* [35–39], the range of detected strain types suggest they may be due to environmental contamination [40].

Naturally uninfected arthropod species like *Ae*.* aegypti* can be amenable to *Wolbachia*-infection through microinjection of the endosymbiont from another insect species into developing embryos [41, 42]. Currently, eight novel strains (*w*AlbA, *w*AlbB, *w*Au, *w*Mel, *w*MelCS, *w*MelPop-CLA, *w*Pip and *w*Ri) have been transinfected into *Ae*.* aegypti* to be evaluated for vector control applications [42–46]. *Aedes aegypti* is widespread in tropical and subtropical regions globally [47, 48], and since its detection in California in 2011 it has become widespread in southern California and the Central Valley [49, 50]. One example of where *Wolbachia*-infected *Ae*.* aegypti* males have been used to reduce mosquito populations through cytoplasmic incompatibility is through the DeBug Fresno California programme, which released male *Ae*.* aegypti* with the *w*AlbB strain of *Wolbachia* to reduce local *Ae*.* aegypti* populations [34]. A second method of using *Wolbachia*-infections for mosquito control relies on the introduction of *Wolbachia-*infected male and female mosquitoes to replace uninfected mosquito populations [12, 51, 52]. *Aedes aegypti* populations (each with a unique *Wolbachia* strain, *w*Mel or *w*AlbB), have been introduced into regions of Australia and Malaysia, respectively [12, 52, 53]. Both strains were shown to reduce the incidence of dengue virus infections [53, 54].

Each *Wolbachia* strain has particular biological characteristics when moved into another vector, and identification of strains is key. Supergroups are used to differentiate major phylogenetic subdivisions within *Wolbachia pipientis* [55]. The *16S* rRNA gene and the *Wolbachia*-surface protein (*wsp*) have been used to characterize supergroups [55, 56]. Within supergroups, *Wolbachia* strains are identified and can be characterized by multilocus sequence typing (MLST) which relies on five conserved bacterial housekeeping genes (*gat*B, *cox*A, *hcp*A, *fts*Z and *fbp*A). Strains are commonly characterized based on the host species in which they are first identified [55, 57, 58]. For instance, *w*Pip is the strain of *Wolbachia* which was identified from the *Culex pipiens* mosquito species. Mosquitoes can be singly or superinfected with more than one *Wolbachia* strain, or infected with multiple variants of the same strain [31].

Merced County is located in the Central Valley of California and includes a diverse range of habitats and mosquito species. While previous studies have identified the presence or absence of *Wolbachia* within some mosquito species throughout California using traditional PCR [58], the current infection status for species in the Central Valley of California and Merced County is unknown. *Wolbachia*-infected mosquitoes as a method of mosquito control has great potential globally, and this vector-control method continues to be developed and refined.

The objectives of this study were to determine the presence or absence of *Wolbachia* in twelve mosquito species collected throughout Merced County, and to characterize the *Wolbachia* supergroup and strain for species with detections. Our study expands current knowledge of *Wolbachia* presence in mosquitoes in Merced and in the Central Valley of California, and would aid in the design of future *Wolbachia*-based mosquito control applications.

## Methods

### Mosquito collections

Adult mosquitoes were collected weekly from June to September, in both 2017 and 2018, using Encephalitis Vector Survey (EVS) traps (Bioquip, Rancho Dominguez, CA, USA) in Merced County. Traps sites were selected for habitats known to harbor the different species of mosquitoes. The EVS traps contained (1–2 kg) of dry ice (carbon dioxide) per container as an attractant for host-seeking female mosquitoes. Traps were hung on trees or fences in close proximity to a water source. The GPS coordinates of the site were recorded using a Garmin etrex High Sensitivity GPS unit (Garmin Ltd., Olathe, KS, USA). Traps were placed during the early afternoon and retrieved the following morning. Samples were transported on ice to a – 20 °C laboratory freezer. Adults were identified on a cold plate using a taxonomic key specific to Californian mosquitoes [59] and stored in 1.5 ml Eppendorf tubes until DNA extraction.

*Aedes aegypti* larvae were collected in addition to adults from several sites in Merced, California. Larvae were collected from water sources, transported to the lab, and reared at laboratory temperature in a BugDorm (MegaView Science, Taichung, Taiwan). Emerged adults were stored at – 20 °C and identification was confirmed using a taxonomic key. A map of trapping locations was constructed for 2017 and 2018 using qGIS v3.8.3-Zanzibar [60]. The Census TIGER/Line file for Merced County, California was retrieved (www.census.gov/cgi-bin/geo/shapefiles2010/main), and site location GPS coordinates were overlaid on the county map.

### DNA extraction

The whole body of the mosquito was used for individual extractions of genomic DNA using the Qiagen DNeasy Blood and Tissue Kit (Qiagen Inc., Valencia, CA, USA), following the manufacturer’s protocol for tissue extraction with a 2 h incubation at 65 °C [61]. Extracted samples were stored at – 20 °C. The DNA quantity was measured using the Qubit® dsDNA HS Assay kit (Life Technologies, Carlsbad, CA, USA). The quantity of DNA in the samples averaged 10–15 ng/µl.

### Screening samples for *Wolbachia *and relative *Wolbachia* density determination

Presence or absence of *Wolbachia* was determined by amplicon detection of the *Wolbachia-*specific *16S* rRNA gene and the general *Wolbachia* surface protein (*wsp*) *via* qPCR in individual field-collected mosquitoes. For each mosquito species collected, a subset of individuals was screened for *Wolbachia*, and individuals tested came from multiple sites or collection dates (Additional file 1: Table S1). The primer combinations for the *Wolbachia*-*16S* rRNA gene and *Wolbachia-*surface-protein (*wsp*) used in our assays are detailed on Table 1. The qPCR cycling conditions were those recommended for the master mix and consisted of holding at 95 °C for 10 min and 40 cycles of 15 s at 95 °C and 1 min at 60 °C. A melt curve stage at the end of the reaction was included. Each sample was analyzed in duplicates (technical replicates), and a non-template control was included. The qPCR assays were run on Applied Biosystems 5700 Fast Real-time PCR (Applied Biosystems, Foster City, CA, USA).

**Table 1:** Primer sequences used for diagnostic testing of *Wolbachia*

Test	Gene target	PCR product (bp)	Primer name	Sequence (5ʹ-3ʹ)	References
*Wolbachia* presence	*16S* rRNA	438	W16S-F	CATACCTATTCGAAGGGATAG	[56, 95, 96]
			W16S-R	TTGCGGGACTTAACCCAACA	
	*wsp* (General)	185	*wsp*-F	GCATTTGGTTAYAAAATGGACGA	[97]
			*wsp*-R	GGAGTGATAGGCATATCTTCAAT	
*Wolbachia* density	*RpS3*	70	RpS3-F	AGCGTGCCAAGTCGATGAG	[98]
			RpS3-R	ACGTACTCGTTGCACGGATCTC	
Supergroup A/B identification	*wsp* (Supergroup A)	556	136F	TGAAATTTTACCTCTTTT	[65]
			691R	AAAAATTAAACGCTACTCCA	
	*wsp* (Supergroup B)	442	81F	TGGTCCAATAAGTGATGAAGAAAC	
			522R	ACCAGCTTTTGCTTGATA	
Multilocus sequence typing	*gatB*	396	gatB_F1	GAKTTAAAYCGYGCAGGBGTT	[57]
			gatB_R1	TGGYAAYTCRGGYAAAGATGA	
	*coxA*	402	coxA_F1	TTGGRGCRATYAACTTTATAG	
			coxA_R1	CTAAAGACTTTKACRCCAGT	
	*hcpA*	444	hcpA_F1	GAAATARCAGTTGCTGCAAA	
			hcpA_R1	GAAAGTYRAGCAAGYTCTG	
	*ftsZ*	435	ftsZ_F1	ATYATGGARCATATAAARGATAG	
			ftsZ_R1	TCRAGYAATGGATTRGATAT	
	*fbpA*	429	fbpA_F1	GCTGCTCCRCTTGGYWTGAT	
			fbpA_R1	CCRCCAGARAAAAYYACTATTC	

The relative *Wolbachia* density was determined *via* qPCR for two species, *Culex pipiens* and *Culex stigmatosoma*. Relative density was determined by measuring the signal amplifications of the *Wolbachia 16S* rRNA or *wsp* gene and the respective reference gene for each mosquito species. The *RpS3* gene was used as a reference gene and primers specific for this location (Table 1) were employed to compare *Wolbachia* densities in the collected samples. The *RpS3* gene is known to be a single copy gene in mosquitoes [62] and is described to be highly conserved [63]. *Culex pipiens* is known to be naturally infected with *Wolbachia* was used as a control to compare the relative *Wolbachia* density to *Cx*.* stigmatosoma*. Samples were compared and the data was analyzed post-run using the $\mathrm{\Delta \Delta }$ Ct method [64]. Data were evaluated using the GraphPad Prism 8.4.2 statistical software, comparing the two species using Studentʼs t-test.

### Determination of *Wolbachia* supergroups

A subset of samples that screened positive for *Wolbachia* by qPCR were used to characterize the *Wolbachia* supergroup. Samples were run using the *Wolbachia wsp* supergroup A and *wsp* supergroup B primers [65] (Table 1). Polymerase chain reaction (PCR) was performed using a mixture of 2 µl of DNA, 1 µl of each forward and reverse primer at 10 µM concentration, 1 µl of Taq polymerase, 5 µl of buffer, 1 µl of dNTPs (2.5 µM) (Takara-Clonetech Bio, Mountain View, CA, USA) and 40 µl of sterile water to make the reaction volume of 51 µl. The temperature profile for *wsp* amplification was the following: initial denaturation for 3 min at 95 °C, followed by 35 cycles of 1 min at 94 °C, 1 min at 55 °C, and 1 min at 72 °C, a final elongation of 10 min at 72 °C and a final hold at 4 °C, modified from the protocol in Zhou et al. [65]. Amplification was confirmed by visualizing products on an agarose gel. Products were purified using USB Exo-sap-it® (Affymetrix Inc., Santa Clara, CA, USA) PCR cleanup kit. Each forward and reverse sequence reaction was prepared using 1 µM primers, 2 µl purified water, and 10 µl purified PCR product per reaction and sequenced on an Applied Biosystems 3730xl DNA Analyzer at the UC Berkeley DNA Sequencing Facility. Multilocus sequence typing (MLST) was also used to characterize supergroups (described below).

### Strain characterization by multilocus sequence typing (MLST)

Species with samples which were successfully sequenced for supergroup A or B were also sequenced by multilocus sequence typing (MLST) using the standard primers for five ubiquitous bacterial housekeeping genes: *cox*A, *gat*B, *fts*Z, *fbp*A and *hcp*A [57] (Table 1). The PCR mix for each gene used a mixture of 2 µl of DNA, 1 µl of each forward and reverse primer at 10 µM concentration, 1 µl of Taq polymerase, 5 µl of buffer, 1 µl of dNTPs (2.5 µM) (Takara-Clonetech Bio Inc., Mountain View, CA, USA) and 40 µl of sterile water to make the reaction volume of 51 µl. The PCR temperature profile for four of the genes (*cox*A, *gat*B, *fts*Z and *hcp*A) was the following: initial denaturation for 2 min at 94 °C, followed by 37 cycles of 30 s at 94 °C, 45 s at 54 °C, and 1.5 min at 72 °C, a final elongation for 10 min at 72 °C and a final hold at 4 °C [57]; the PCR program for the *fbp*A gene was identical except the annealing was for 45 s at 59 °C. PCR amplification was visually confirmed on agarose gels, products purified by USB Exo-sap-it®, and sequencing reactions were similar to those previously described.

### Sequence analysis

*Wolbachia* surface protein (*wsp*) and the MLST genes (*cox*A, *gat*B, *fts*Z, *hcp*A and *fbp*A*)* sequence files were viewed, edited, and aligned in Geneious Prime 2020.05. Consensus sequences were generated and exported as FASTA files. Consensus sequences were queried using the BLASTn program to find sequences with the highest similarity.

*Wolbachia* supergroup sequences from this study were combined with high similarity sequences from GenBank and others from a study by Carvajal et al. [38] to produce a Neighbor-Joining tree. Included in the tree were consensus sequences of 18 samples from this study [*Cx*.* pipiens* (*n* = 8) *Culex stigmatosoma* (*n* = 9) and *Culiseta inornata* (*n* = 1)] and an additional 15 *wsp* sequence files from GenBank which represented 11 genera previously confirmed with detections of *Wolbachia*. The species selected for comparison were *Aedes albopictus* (AF020058, AF020059), *Brugia malayi* (AJ252061), *Culex pipiens* (AF020061), *Culex quinquefasciatus* (AF020060), *Dirofilaria imitis* (AJ252062), *Drosophila melanogaster* (AF020072), *Drosophila simulans* (AF020070), *Glossina austeni* (AF020077), *Glossina morsitans* (AF020079), *Muscidifurax uniraptor* (AF020071) and *Phlebotomus papatasi* (AF020082) [38], and three additional sequences (*Loxoblemmus* sp. MG97910, *Myrmecophilus* sp. MK995471 and *Cerapachys augustae* KC137155) of high similarity. These 33 sequences were subjected to multiple sequence alignment using the ClustalW algorithm in MEGA 7.0. The Gamma distributed, Tamura 3-parameter substitution model was chosen based on the lowest Bayesian information criterion. A Neighbor-Joining tree was constructed using 1000 bootstraps in MEGA 7.0 [66].

*Wolbachia* strains were characterized by concatenating the *cox*A, *gat*B, *fts*Z, *hcp*A and *fbp*A gene sequences from each sample in Geneious. Following concatenation, each sequence was exported in FASTA format and queried against the *Wolbachia* MLST database (https://pubmlst.org/Wolbachia/) to determine allelic profiles [57, 67]. An exact match with the queried database was necessary to distinguish profile composition. All sequences were submitted to Genbank.

## Results

### Mosquito collections, identification and abundance

**Fig. 1:**
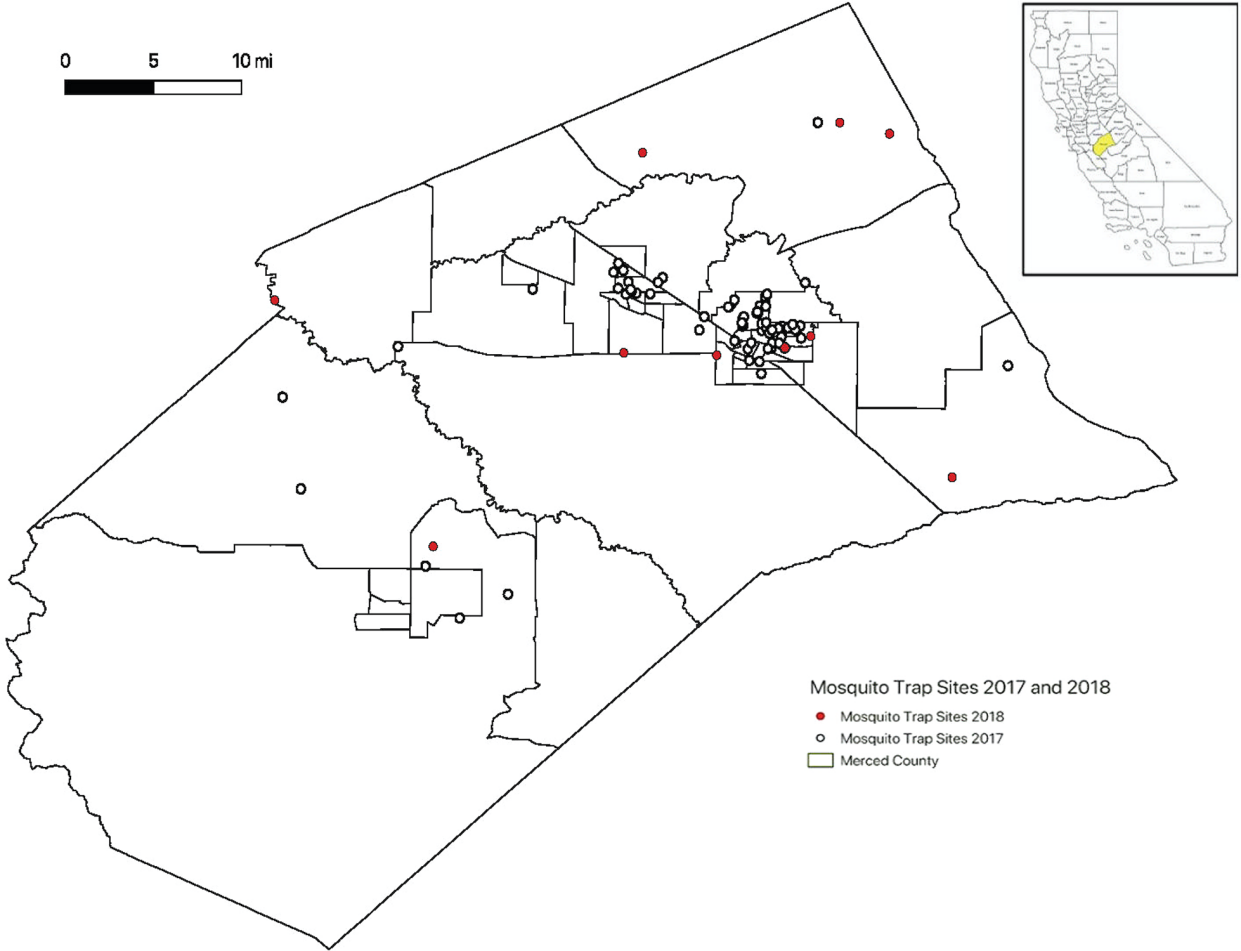
Merced County mosquito trap sites. Collections made in 2017 are indicated with white circles, and collections in 2018 are indicated with red circles

In total, 12 mosquito species from 4 genera were collected from 85 sites within Merced county in 2017 and 2018 (Table 2, Additional file 1: Table S1). There was a total of 7150 mosquitoes identified to species. The species collected were the following: *Aedes melanimon*, *Aedes vexans*, *Aedes nigromaculis*, *Aedes aegypti*, *Culex stigmatosoma*, *Culex pipiens*, *Culex tarsalis*, *Anopheles franciscanus*, *Anopheles freeborni*, *Anopheles punctipennis*, *Culiseta incidens*, *Culiseta inornata* (Table 2). These species represent the diversity of nearly every mosquito from the region where trapping occurred [59]. The 85 trap sites were in the vicinity of 8 cities within Merced county: Atwater, Ballico, Hilmar, Le Grand, Los Banos, Merced, Snelling and Winton (Fig. 1, Table 2). Each mosquito species was trapped from two to five different regions of the county (Table 2, Additional file 1: Table S1), to provide geographic diversity in samples which were tested. Some mosquito species were more abundant than others. For example, *Cx*.* pipiens* and *Cx*.* tarsalis* were trapped in cities as well as in rural sites (Additional file 1: Table S1). *Ae*.* melanimon* and *Ae*.* vexans* adults were most abundant within rural wetland habitats. *Aedes aegypti* was found in several Merced neighborhoods and near the Merced Zoo. *Anopheles franciscanus*, *An*.* freeborni* and *An*.* punctipennis* were found at rural riparian sites. *Culex stigmatosoma* were numerous at a semi-natural rural site near dairy runoff. *Aedes nigromaculis*, *Cs*.* incidens* and *Cs*.* inornata* were collected from rural and residential properties.

**Table 2:** Mosquito species collected and screened for *Wolbachia* by qPCR of *16S* rRNA gene and *WSP*

Mosquito species	Total trapped	Atwater	Ballico	Hilmar	Le Grand	Los Banos	Merced	Snelling	*wsp*^a^	*16S*^a^	Total^b^
*Ae*. *melanimon*	1827	–	–	5/26	–	1/20	–	0/9	4/55	5/55	6/55 (10.9%)
*Ae*. *nigromaculis*	12	–	0/1	-	–	− 0/8	0/3	–	–	–	0/12 (0%)
*Ae*. *vexans*	488	–	–	2/36	–	0/16	–	–	0/52	2/52	2/52 (3.9%)
*Ae*. *aegypti*	60	–	–	–	–	–	0/60	–	–	–	0/60 (0%)
*Cx*. *pipiens*	994	5/5	15/15	–	–	–	10/10	1/7	31/37	31/37	31/37 (83.8%)
*Cx*. *stigmatosoma*	36	2/2	28/28	–	–	–	0/1	0/3	30/34	30/34	30/34 (88.2%)
*Cx*. *tarsalis*	3878	–	1/15	–	0/5	–	0/4	0/2	0/26	1/26	1/26 (3.9%)
*An*. *franciscanus*	2	–	–	–	–	–	–	0/2	–	–	0/2 (0%)
*An*. *freeborni*	221	–	0/29	0/1	0/22	–	0/1	0/7	–	–	0/60 (0%)
*An*. *punctipennis*	19	–	–	–	–	0/1	–	1/18	0/19	1/19	1/19 (5.3%)
*Culiseta* *incidens*	94	–	–	–	0/1	–	1/35	0/6	1/42	1/42	1/42 (2.4%)
Total	7150	7/7	44/88	7/63	0/28	1/45	11/114	3/60	67/406	72/406	73/406

^a^Number positive/Number tested

^b^Percent of samples screened positive for *Wolbachia* by either *wsp* or *16S* rRNA. Collections details for all mosquitoes are detailed in Additional file 1: Table S1

### *Wolbachia* screening with qPCR

For each species, 30–50 mosquitoes were typically screened for the presence or absence of *Wolbachia*, except for a few species which had smaller numbers of individuals collected (Table 2). A total of 406 mosquitoes were screened for *Wolbachia* prevalence using qPCR, and all mosquitoes screened were females. *Wolbachia* was detected within 73 of the 406 samples tested, and sites with mosquitoes positive for *Wolbachia* were found throughout the county (Table 2, Additional file 1: Table S1). Eight species within four genera tested positive for *Wolbachia* (Table 2). The frequency and percent of samples positive for each species from highest to lowest was the following: *Cx*.* stigmatosoma* (30/34; 88.2%), *Cx*.* pipiens* (31/37; 83.8%), *Cs*.* inornata* (1/7; 14.3%), *Ae*.* melanimon* (6/55: 10.9%), *An*.* punctipennis* (1/19: 5.3%), *Cx*.* tarsalis* (1/26: 3.9%), *Ae*.* vexans* (2/52; 3.9%) and *Cs*.* incidens* (1/42; 2.4%) (Table 2). Species where no *Wolbachia* was detected were *An*.* freeborni*, *An*.* franciscanus*, *Ae*.* nigromaculis* and *Ae*.* aegypti* (Table 2).

Each species was screened by qPCR for *Wolbachia* with two primers. For *Cx*.* pipiens* and *Cx*.* stigmatosoma*, all individuals were positive for *Wolbachia* when tested with both genes (*16S* rRNA and *wsp*) (Table 2). In a few cases, one primer would detect *Wolbachia*, while another would not (Table 2). For *Ae*.* melanimon*, *Cx*.* tarsalis*, *Cs, incidens*, *Cs. inornata*, *An. punctipennis* and *Ae. vexans*, *Wolbachia* was detected in very few individuals (Table 2). For *Cs. incidens* and *Cs. inornata*, both primers detected only one positive individual (Table 2). Six individuals were positive detections with the *16S* rRNA primer set but were negative with *wsp* (one *An. punctipennis*, one *Cx. tarsalis*, two *Ae. vexans* and two *Ae. melanimon).* Only one sample was negative with *16S* rRNA but positive for *wsp* (*Ae. melanimon*) (Table 2, Additional file 1: Table S1).

To evaluate the relative *Wolbachia* density of the two *Culex* spp., we conducted a relative comparison using qPCR for 30 individuals each of *Culex stigmatosoma* and *Cx. pipiens*, the later which was used as a control. The relative *Wolbachia* density comparison indicated no significant difference between the two species (*16S*, t-test, *t* = 0.80, *df* = 48, *P* = 0.43; *wsp*, t-test, *t* = − 1.34, *df* = 48, *P* = 0.18).

### *Wolbachia* supergroup identification

**Fig. 2:**
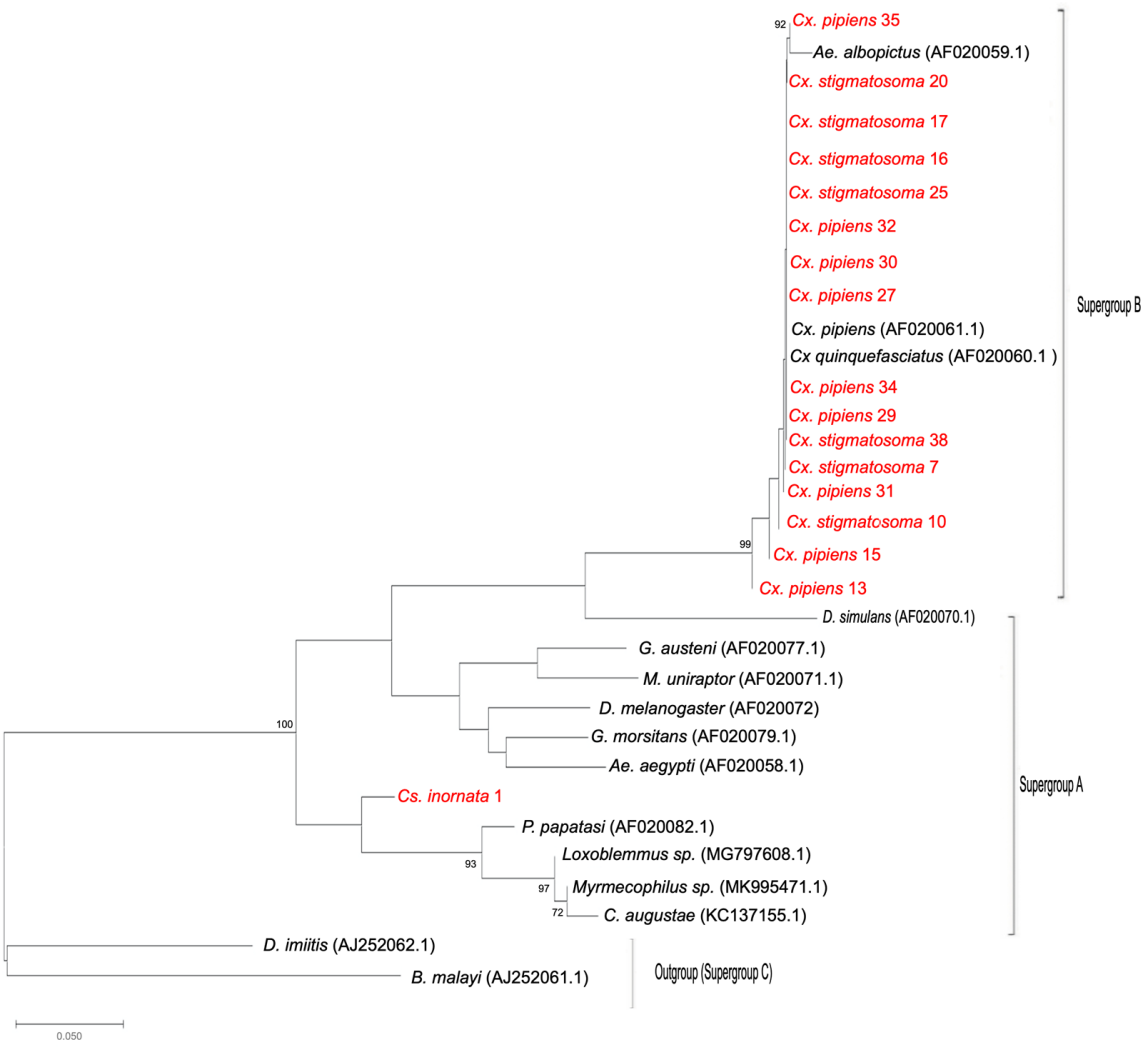
Neighbor-joining tree for *Cx*. *pipiens*, *Cx*. *stigmatosoma* and *Cs*. *inornata* (this study) combined with 15 *Wolbachia-*surface-protein (*wsp*) supergroup A and B sequences from GenBank and Carvajal et al. [38]. Sequences from this study are shown in red. The tree was based on the Tamara 3-parameter, Gamma distributed model with 1000 bootstrap replications

*Wolbachia* supergroup identification was carried out by PCR of samples using general *wsp* supergroup A and supergroup B primers. A total of 18 *Wolbachia* surface protein sequences were generated from three species, *Cx. pipiens* (*n* = 8), *Cx. stigmatosoma* (*n* = 9) and *Cs. inornata* (*n* = 1). *Wsp* sequences were not successfully obtained from the other species with low frequency *Wolbachia* detections (Table 2). The sequences produced in this study were combined with an additional 15 *wsp* sequences from GenBank for supergroup comparison (described above). The *Cx. pipiens* and *Cx. stigmatosoma* individuals grouped with the reference supergroup B samples, and *Cs. inornata* grouped with supergroup A reference samples (Fig. 2).

### *Wolbachia* strain characterization

There were five individual *Cx. pipiens* which had 5 MLST genes (*cox*A, *gat*B, *fts*Z, *hcp*A and *fbp*A*)* successfully sequenced (*Cx. pipiens* nos. 29, 31, 32, 34 and 35) and they were matches with strain type 9, *w*Pip supergroup B *Wolbachia* in the MLST database (Table 3). Four additional *Cx. pipiens* were similar at 3 or 4 of the five gene sequences to strain type 9 *w*Pip; however, these had a low quality *hcp*A sequences and exact match of that allele could not be confirmed.

**Table 3:** Multilocus sequence typing (MLST) to identify *Wolbachia* strains

Species	Sample #	MLST gene						Strain	SG^a^	Strain #
		*gat*B	*cox*A	*hcp*A		*fts*Z	*fbp*A			
*Cx*. *pipiens*	*Cxpip* 29	4	3	354		22	444	*w*Pip	B	9
	*Cxpip* 31	4	3	354		22	444	*w*Pip	B	9
	*Cxpip* 32	4	3	354		22	444	*w*Pip	B	9
	*Cxpip* 34	4	227	354		22	444	*w*Pip	B	9
	*Cxpip* 35	4	3	354		22	444	*w*Pip	B	9
*Cx*. *stigmatosoma*	*Cxstig* 15	4	3	354		22	444	*w*Pip	B	9
	*Cxstig* 10	148	3	354		22	444	*w*Pip	B	9

^a^Supergroup

For *Cx. stigmatosoma,* two samples had complete gene sequences for the five MLST genes (*cox*A, *gat*B, *fts*Z, *hcp*A and *fbp*A*)*; the allelic profile for *Cx. stigmatosoma* samples 10 and 15 from Ballico were a match for the five sequences retrieved from several *Cx. pipiens* samples (nos. 29, 31, 32, and 35), and these were characterized as *Wolbachia w*Pip supergroup B strain-type 9 (Table 3). The two *Cx. stigmatosoma* individuals had four of the five MLST genes sequenced and also matched strain type 9, but only partial sequences were obtained for the *hcp*A gene. The *hcp*A locus has been observed with variable sequence lengths, ranging from 435 to 477 bp (pubmlst.org/*Wolbachia*). Five additional samples (*Cx. stigmatosoma* nos. 16,17, 20, 25, 38) were sequenced at 3 or 4 of the 5 genes, which also had matching profiles to *Wolbachia* housekeeping genes (*cox*A, *fts*Z and *fbp*A) from this study.

One *Cs. inornata* sample had a detection of *Wolbachia* with *16S* rRNA gene, and this individual was used to generate sequence data for the five MLST genes. The one *Cs. inornata* had four sequences (*fbp*A, *gat*B, *cox*A, *ftsz*) which had mlst allele matches; these sequences matched *fbp*A allele 277, *gat*B 312, *cox*A 236, and *ftsz* 154, while *hcp*A had no match [67]. For *Cs. inornata*, the *wsp* sequence grouped with others in supergroup A.

## Discussion

This study screened 12 field-collected mosquito species in the Central Valley of California for the presence or absence of *Wolbachia*, and for species with *Wolbachia* detections, attempted to characterize the supergroup and strain type. The 12 mosquito species identified and screened were the following: *Ae. melanimon*, *Ae. nigromaculis*, *Ae. vexans*, *Ae. aegypti*, *Cx. pipiens*, *Cx. stigmatosoma*, *Cx. tarsalis*, *An. franciscanus*, *An. freeborni*, *An. punctipennis*, *Cs. incidens* and *Cs. inornata. Wolbachia* was detected in eight of the mosquito species. To our knowledge, this study is the first to report *Wolbachia* detection in five of these species (*Ae. melanimon*, *Cx. stigmatosoma*, *Cx. tarsalis*, *Cs. incidens* and *Cs. inornata*), while three species which were positive in this study have been previously reported in the literature (*Ae. vexans*, *Cx. pipiens* and *An. punctipennis*). The *Wolbachia* supergroup was determined for two of these new records (*Cx. stigmatosoma* and *Cs. inornata*), and the strain was characterized for *Cx. stigmatosoma* using MLST. The other species with detections of *Wolbachia* had a very low prevalence (frequency) and could not be sequenced.

The two mosquito species which were positive for *Wolbachia* at high frequencies (prevalence) were *Cx. pipiens* and *Cx. stigmatosoma*. The other six species showed detections of *Wolbachia* at low prevalence (< 13%). Furthermore, when the relative *Wolbachia* density was compared between *Cx. pipiens* and *Cx. stigmatosoma*, there was no statistical difference indicating that these two species potentially hold similar *Wolbachia* densities. Further assessment *via* absolute quantification of *Wolbachia* would further confirm this finding. In addition, future work with *Cx. stigmatosoma* could investigate maternal transmission to provide supporting evidence for *Wolbachia* infection. The inability to sequence *Wolbachia* in the species with low *Wolbachia* prevalence could be due to a low *Wolbachia* density. One species, *Cs. inornata*, had a low *Wolbachia* prevalence (13%), yet the *wsp*A sequence was obtained which allowed it to be tentatively classified into supergroup A. Four of five MLST genes were sequenced for *Cs. inornata* in this study. This *Wolbachia* isolate may potentially represent a new *Wolbachia* strain, but further research would be needed with additional samples collected to replicate detection of *Wolbachia*.

*Wolbachia* infections were previously reported in *Cx. pipiens* [58, 68]*, An. punctipennis* [69], and *Ae vexans* [70]. Although *Wolbachia* has been previously detected in *An. punctipennis* and *Ae. vexans*, currently there is no description of a strain type for these mosquitoes. Our study did not detect *Wolbachia* in several mosquito species including *An. freeborni*, *An. franciscanus*, *Ae. nigromaculis* and *Ae. aegypti*. Although a few studies have indicated *Wolbachia* detection in *Ae. aegypti* [36–39], others found absence of infection in this species [40, 71] and suggest that the variability of strains found in previous studies on *Ae. aegypti* may indicate environmental contamination rather than a true *Wolbachia* infection. Ross et al. [40] recommend that to confirm *Wolbachia* infection, experiments should be run to demonstrate maternal transmission or to visualize *Wolbachia* in the mosquito using a method such as florescent *in situ* hybridization (FISH), in addition to determining sequences. *Culex pipiens* is well known for its infection with *Wolbachia*, as *Wolbachia pipientis* was first described from this mosquito species [1, 2]. Previous research identified *w*Pip supergroup B infections in the *Cx. pipiens* species complex in five California populations [58]. Since then more than 60 *w*Pip haplotypes have been identified [31, 72]. Our study screened *Cx. pipiens* from four sites and found individuals from all sites carrying *Wolbachia*. In the present study, the MLST results for *Cx. pipiens* found strain type 9 supergroup B among samples with complete allelic profile data. These were all acquired from the Ballico collection site. Isolates of strain type 9 have been documented in *Cx. pipiens* and *Cx. quinquefasciatus* [67]. Other studies have found *Cx. pipiens* with strain type 9 in Placer County, California and Tompkins County, New York; while *Wolbachia*-infected *Cx. quinquefasciatus* were found in Hawaii, Midway and Kenya [57, 73].

Interestingly, our study also found a new *Wolbachia* detection record for *Cx. stigmatosoma*. This species is highly ornithophilic [74] and often found in urban residential areas and near farms. It prefers foul water sources like street drains and dairy lagoons for oviposition [75]. These types of habitat are similar to those where *Cx. pipiens* can also be found. This species is known to occur throughout the western USA to Mexico, Central America and northern South America [75, 76]. *Culex stigmatosoma* is a competent vector of West Nile virus, and is capable of transmitting St Louis encephalitis and avian malaria [76, 77].

*Culex stigmatosoma* had MLST sequences produced from two different collection sites. One site was a rural semi-natural habitat near a dairy (Ballico), and another was a rural farm in Atwater. At the first site, *Cx. stigmatosoma* had sequences from the five MLST genes that were an identical match for those from *Wolbachia* strain type 9 (ST-9) *w*Pip infection in the MLST database, sequences which were identical to those characterized from *Cx. pipiens* tested in this study (Table 3). Although the five MLST housekeeping genes sequenced from *Cx. stigmatosoma* matched those of *Cx. pipiens* for strain type 9 *w*Pip, it would be worthwhile to examine differences in *Wolbachia* from these two species using a more comprehensive method such as comparative genomics before concluding the two species harbor the same strain [78]. Bleidorn & Gerth (2018) discussed the limits of the MLST for *Wolbachia* strain characterization; one of these is that several of the MLST genes used to characterize *Wolbachia* strains evolve slowly, and may not sufficiently differentiate among strains where significant biological differences may exist. In this study, *Cx. stigmatosoma* was not likely to be misidentified as adult *Cx. pipiens*. Adult *Cx. stigmatosoma* more closely resemble *Cx. tarsalis*, but the two species are distinguished by distinct markings on ventral abdominal segments [59]. *Culex stigmatosoma* had a high prevalence (frequency) of individuals with detections of *Wolbachia*. The second collection site (Atwater) where *Cx. stigmatosoma* was positive for *Wolbachia* in this study also had an individual with MLST alleles match those of *Cx. pipiens Wolbachia* strain type as well (strain 9). This species could represent a new *Wolbachia* infection, not just a detection of *Wolbachia*. However, further studies would be needed to provide evidence of infection which are complementary to sequencing, such as FISH or loop mediated isothermal amplification (LAMP) [40].

The *Wolbachia* similarity observed between the two species above (*Cx. pipiens* and *Cx. stigmatosoma*) is not unusual. In fact, several studies have documented high similarity among some *Wolbachia-*infections in hosts within the same genus [57, 79, 80]. In Italy, evidence of natural *w*Pip *Wolbachia* infections have been identified within *Culex modestus* and *Culex torrentium* mosquito species, and there was no observable divergence in *wsp* sequences when compared to field collected *Cx. pipiens* [79]. Another example was documented in Portugal, where low prevalence *Wolbachia*-infections were found in *Culex theileri* and indistinguishable from *Cx. pipiens* by *16S* rRNA, *ank2* and *pk1* genes [80]. Furthermore, the results of restricted fragment length polymorphisms (RFLP) suggested a shared *w*Pip haplotype I infection among both *Cx. theileri* and *Cx. pipiens*. Thus, it is not surprising that two closely related *Culex* species in the present study could harbor very similar or closely related *Wolbachia* strains.

*Culiseta inornata* had several MLST genes match those in the MLST database. When grouped in the supergroup phylogeny with other vector species, *Cs*.* inornata* was closely related to supergroup A infections previously reported in a dipteran, *Phlebotomos papatasi* (sand fly) and an orthopteran (*Loxoblemmus* spp.) (Fig. 2).* Culiseta inornata* in this study was collected in a semi-natural riparian habitat along the Merced River. This species is predominant in rural areas, and is capable of vectoring West Nile virus, western equine encephalitis, St Louis encephalitis, Japanese encephalitis, California encephalitis and avian malaria [76, 81, 82]. This species occurs throughout the United States, with a known presence in 46 states from California to New York and the range also expands north into Canada [75, 83]. *Culiseta inornata* persists through the winter months, which could have implications for the seasonality of arbovirus transmission. Given that *Cs*.* inornata* transmits a number of vector-borne diseases, further study to investigate *Wolbachia* within this species could be worthwhile, since *Wolbachia* can influence vector competence. Moreover, future research could investigate whether *Wolbachia* in this species persist within other populations in California or other regions.

Several other mosquito species had *Wolbachia* at a low frequency or density. Some of these species have been previously tested through traditional PCR, but perhaps escaped detection due to the lower sensitivity of traditional PCR compared to qPCR [58]. Our study detected *Ae*.* melanimon* with *Wolbachia* at a low frequency. This study is the first record of *Ae*. *melanimon* with detection of *Wolbachia*, but additional tests as previously described would be needed to confirm infection [40]. *Aedes melanimon* are widely distributed throughout western and southwestern USA and Canada [75, 84, 85]. This species prefers to oviposit in or around irrigated pastures, ponds and fields. *Aedes melanimon* is the primary vector of California encephalitis and is capable of transmitting western equine encephalitis and West Nile virus [76, 86, 87]. Past literature has identified *Ae*.* melanimon* to have a secondary role in maintenance of western equine encephalitis virus within the Central Valley of California, and has identified this mosquito as preferentially feeding on humans and other mammals [88, 89]. Along with *Ae*.* melanimon*, several other species (*An*.* punctipennis*, *Cx*.* tarsalis* and *Cs*. *incidens*) had very low prevalence (all less than 10%), perhaps due to horizontal transmission [90]. Recently, Shaikevich et al. [90] suggested that *Wolbachia* diversity is likely attributed to horizontal transfer and strain recombination. By utilizing one-allele-criterion (OAC) phylogenetic networks, the authors suggest a link between the *Ae*. *albopictus* (*w*AlbB) *Wolbachia* strain and *Wolbachia* from ants; furthermore, that supergroup B strains from mosquitoes are linked with *Wolbachia* from Lepidoptera [90]. Routes of horizontal transmission have been shown to occur through parasitism, shared habitats, and predation [11, 91–93].

## Conclusions

Our survey of *Wolbachia* infections in Merced county mosquitoes identified new *Wolbachia* detections, providing information to support current and future *Wolbachia*-mediated vector control applications. As noted, it will be important to confirm *Wolbachia* detections are true infections by providing evidence in addition to *Wolbachia* sequences. *Wolbachia-*based approaches have been implemented within vector control strategies by propagation of a desired strain within an uninfected population, or by inducing cytoplasmic incompatibility through mating incompatibility. Successful integration depends on the strain chosen for its effects on the novel host [94]. Characterizing new *Wolbachia* strains and determining their mosquito host species are critical to efforts to further develop *Wolbachia*-mediated vector control applications.

## Supplementary information

**Additional file 1: Table S1.** Mosquito collections in Merced county.

## Data Availability

The datasets generated during this study consist of sequences submitted to GenBank under the accession numbers MW125593- MW125610 (*wsp*), MW133153-MW133170 (*ftsz*), MW133171-MW133187 (*cox*A), MW133188-MW133204 (*fbp*A), MW133205-MW133220 (*gat*B), and MW133221-MW133228 (*hcp*A).
