# Supplementary material for: Wolbachia in mosquitoes from the Central Valley of California, USA
Source: Parasit Vectors. 2020 Nov 10;13:558. doi: 10.1186/s13071-020-04429-z (PMC7653878; doi:10.1186/s13071-020-04429-z)
Supplement: Supplementary file 1 — Additional file 1: Table S1. Mosquito collections in Merced county. [file 13071_2020_4429_MOESM1_ESM.docx]

| **Additional file 1: Table S1.** Mosquito collections in Merced county. *Wolbachia* positive per number tested (#Pos/#test) | | | | | |  |  |  |
| --- | --- | --- | --- | --- | --- | --- | --- | --- |
|  |  |  |  |  |  |  |  |  |
| **Species Name** | **Site Name** | **Trap Date** | **GPS**  **Coord** | **# Caught** | **Samples Extracted** | **#Pos/#test**  **qPCR wsp** | **#Pos/**  **#test**  **qPCR 16s** | **Tot#pos/ tested** |
| *Aedes melanimon* |  |  |  |  |  |  |  |  |
|  | Snelling Rural | Jun. 5  2018 | 37.5108, -120.34821 | 3 | *Ae. melanimon* 86-88 | 0/3 | 0/3 | 0/3 |
|  |  |  |  |  |  |  |  |  |
|  | Snelling Rural | Jun. 12,  2018 | 37.51078, -120.34822 | 5 | *Ae. melanimon* 89-93 | 0/5 | 0/5 | 0/5 |
|  |  |  |  |  |  |  |  |  |
|  | Snelling Rural | Jun. 12 | 37.50989, -120.34576 | 1 | *Ae. melanimon* 94 | 0/1 | 0/1 | 0/1 |
|  |  |  |  |  |  |  |  |  |
|  | Los Banos Rural | Aug. 28 | 37.107600, -120.811800 | 474 | *Ae. melanimon* 25-34 | 0/10 | 1/10 | 1/10 |
|  |  |  |  |  |  |  |  |  |
|  | Hilmar Rural | Sept. 4 | 37.352853, -120.97421 | 92 | *Ae. melanimon* 35-54 | 3/16 | 4/16 | 4/16 |
|  |  |  |  |  |  |  |  |  |
|  | Hilmar Rural | Sept. 4 | 37.352852, -120.974157 | 56 | *Ae. melanimon* 95-104 | 1/10 | 0/10 | 1/10 |
|  |  |  |  |  |  |  |  |  |
|  | Los Banos Rural | Sept. 18 | 37.144572, -120.801012 | 417 | *Ae. melanimon* 105-114 | 0/10 | 0/10 | 0/10 |
|  |  |  |  |  | # screened per gene, combined: | 4/55 | 5/55 | 6/55 |
| *Aedes nigromaculis* | 2017 |  |  |  |  |  |  |  |
|  | Merced Rural Site 5 | 6/26/17 | 37.33740, -120.53511 | 1 | *Ae. nigromaculis* 4 | 0/1 | 0/1 | 0/1 |
|  |  |  |  |  |  |  |  |  |
|  | Gustine Rural Site 2 | 9/17/17 | 37.26207, -120.96567 | 5 | *Ae. nigromaculis* 13-17 | 0/5 | 0/5 | 0/5 |
|  |  |  |  |  |  |  |  |  |
|  | Merced Urban Site 2 | 9/19/17 | 37.35341, -120.47293 | 1 | *Ae. nigromaculis* 19 | 0/1 | 0/1 | 0/1 |
|  |  |  |  |  |  |  |  |  |
|  | Stevinson Ranch Trap 4 | 9/23/17 | 37.29098, -120.82479 | 3 | *Ae. nigromaculis* 1-3 | 0/3 | 0/3 | 0/3 |
| *Aedes nigromaculis* | 2018 |  |  |  |  |  |  |  |
|  | Ballico Rural | Jun. 27 | 37.49215, -120.59813 | 1 | *Ae. nigromaculis* 18 | 0/1 | 0/1 | 0/1 |
|  |  |  |  |  |  |  |  |  |
|  | Merced Rural 2 | Jun. 28 | 37.30101, -120.52242 | 1 | *Ae. nigromaculis* 20 | 0/1 | 0/1 | 0/1 |
|  |  |  |  |  |  |  |  |  |
|  |  |  |  |  | # screened per gene, combined: | 0/12 | 0/12 | 0/12 |
| *Aedes vexans* | 2018 |  |  |  |  |  |  |  |
|  |  |  |  |  |  |  |  |  |
|  | Hilmar Rural | Aug. 16 | 37.352858, -120.97421 | 71 | *Ae. vexans* 1-24 | 0/24 | 1/24 | 1/24 |
|  |  |  |  |  |  |  |  |  |
|  | Hilmar Rural | Sept. 4 | 37.352853, -120.97421 | 20 | *Ae. vexans* 63-74 | 0/12 | 1/12 | 1/12 |
|  |  |  |  |  |  |  |  |  |
|  | Los Banos Rural | Sept. 18 | 37.144572, -120.801012 | 271 | *Ae. vexans* 75-90 | 0/16 | 0/16 | 0/16 |
|  |  |  |  |  | # screened per gene, combined: | 0/52 | 2/52 | 2/52 |
| *Aedes aegypti* |  |  |  |  |  |  |  |  |
|  | Merced Urban Site 37 | 9/1/17 | 37.31273, -120.48699 | 10 | *Ae. aegypti* 21-30 | 0/10 | 0/10 | 0/10 |
|  |  |  |  |  |  |  |  |  |
|  | Merced Urban 38 | 10/5/17 | 37.33027, -120.45603 | 4 | *Ae. aegypti* 41-44 | 0/4 | 0/4 | 0/4 |
|  |  |  |  |  |  |  |  |  |
|  | Merced Urban 39 | 10/5/17 | 37.32911, -120.45455 | 2 | *Ae. aegypti* 31, 45 | 0/2 | 0/2 | 0/2 |
|  |  |  |  |  |  |  |  |  |
|  | Merced Urban Site 38 | 10/9/17 | 37.33027, -120.45603 | 26 | *Ae. aegypti* 1-8, 11-13, 15,16, 18, 48-59 | 0/26 | 0/26 | 0/26 |
|  |  |  |  |  |  |  |  |  |
|  | Merced Urban Site 39 | 10/9/17 | 37.32911, -120.45455 | 14 | *Ae. aegypti* 9,14, 17,19, 20, 32-39, 60 | 0/14 | 0/14 | 0/14 |
|  |  |  |  |  |  |  |  |  |
|  | Merced Urban 39 | 10/10/17 | 37.32911, -120.45455 | 1 | *Ae. aegypti* 40 | 0/1 | 0/1 | 0/1 |
|  |  |  |  |  |  |  |  |  |
|  | Merced Urban 38 | 10/10/17 | 37.33027, -120.45603 | 2 | *Ae. aegypti* 46, 47 | 0/2 | 0/2 | 0/2 |
|  |  |  |  |  |  |  |  |  |
|  | Merced Urban site 39 | 10/11/17 | 37.32911, -120.45455 | 1 | *Ae. aegypti* 10 | 0/1 | 0/1 | 0/1 |
|  |  |  |  |  |  |  |  |  |
|  |  |  |  |  | # screened per gene, combined: | 0/60 | 0/60 | 0/60 |
| *Anopheles freeborni* | 2018 |  |  |  |  |  |  |  |
|  | Le Grand Rural | Jun. 19 | 37.19358, -120.26038 | 9 | *An. freeborni* 90-98 | 0/9 | 0/9 | 0/9 |
|  |  |  |  |  |  |  |  |  |
|  | Ballico Rural | Jun. 27 | 37.49213, -120.59809 | 1 | *An. freeborni* 41 | 0/1 | 0/1 | 0/1 |
|  |  |  |  |  |  |  |  |  |
|  | Ballico Rural | Jun. 27 | 37.49215, -120.59813 | 6 | *An. freeborni* 42-47 | 0/6 | 0/6 | 0/6 |
|  |  |  |  |  |  |  |  |  |
|  | Ballico Rural | Jun. 27 | 37.49248, -120.59816 | 5 | *An. freeborni* 48-52 | 0/5 | 0/5 | 0/5 |
|  |  |  |  |  |  |  |  |  |
|  | Ballico Rural | Jun. 27 | 37.49245, -120.59838 | 10 | *An. freeborni* 53-58 | 0/6 | 0/6 | 0/6 |
|  |  |  |  |  |  |  |  |  |
|  | Ballico Rural | Jun. 27 | 37.49332, -120.59822 | 11 | *An. freeborni* 79-89 | 0/11 | 0/11 | 0/11 |
|  |  |  |  |  |  |  |  |  |
|  | Merced Rural | Jun. 28 | 37.31892, -120.42629 | 1 | *An. freeborni* 100 | 0/1 | 0/1 | 0/1 |
|  |  |  |  |  |  |  |  |  |
|  | Snelling Rural | Jul. 11 | 37.51078, -120.34820 | 4 | *An. freeborni* 59-62 | 0/4 | 0/4 | 0/4 |
|  |  |  |  |  |  |  |  |  |
|  | Snelling Rural | Jul. 11 | 37.51079, -120.34826 | 2 | *An. freeborni* 63, 64 | 0/2 | 0/2 | 0/2 |
|  |  |  |  |  |  |  |  |  |
|  | Snelling Rural | Jul. 11 | 37.51735, -120.34617 | 1 | *An. freeborni* 65 | 0/1 | 0/1 | 0/1 |
|  |  |  |  |  |  |  |  |  |
|  | Le Grand Rural | Jul. 12 | 37.52062, -120.39664 | 3 | *An. freeborni* 66-68 | 0/3 | 0/3 | 0/3 |
|  |  |  |  |  |  |  |  |  |
|  | Le Grand Rural | Jul. 12 | 37.19260, -120.25952 | 3 | *An. freeborni* 69-71 | 0/3 | 0/3 | 0/3 |
|  |  |  |  |  |  |  |  |  |
|  | Le Grand Rural | Jul. 12 | 37.19262, -120.25940 | 8 | *An. freeborni* 72-78 | 0/7 | 0/7 | 0/7 |
|  |  |  |  |  |  |  |  |  |
|  | Hilmar Rural | Aug. 16 | 37.352858, -120.97421 | 1 | *An. freeborni* 99 | 0/1 | 0/1 | 0/1 |
|  |  |  |  |  |  |  |  |  |
|  |  |  |  |  | # screened per gene, combined: | 0/60 | 0/60 | 0/60 |
| *Anopheles franciscanus* | 2018 |  |  |  |  |  |  |  |
|  | Snelling Rural | Jul. 11  2018 | 37.51078, -120.34820 | 1 | *An. franciscanus* 5 | 0/1 | 0/1 | 0/1 |
|  |  |  |  |  |  |  |  |  |
|  | Snelling Rural | Aug. 20  2018 | 37.51078, -120.34828 | 1 | *An. franciscanus* 6 | 0/1 | 0/1 | 0/1 |
|  |  |  |  |  |  |  |  |  |
|  |  |  |  |  | # screened per gene, combined: | 0/2 | 0/2 | 0/2 |
| *Anopheles punctipennis* | 2017 |  |  |  |  |  |  |  |
|  | Snelling Rural Site 2 | 7/12/17 | 37.51111, -120.34744 | 1 | *An. punctipennis* 1 | 0/1 | 0/1 | 0/1 |
|  |  |  |  |  |  |  |  |  |
| *Anopheles punctipennis* | 2018 |  |  |  |  |  |  |  |
|  | Snelling Rural | Jun. 5  2018 | 37.51080, -120.34819 | 2 | *An. punctipennis* 2, 3 | 0/2 | 0/2 | 0/2 |
|  |  |  |  |  |  |  |  |  |
|  | Snelling Rural | Jun. 5  2018 | 37.51080, -120.34821 | 2 | *An. punctipennis* 4, 5 | 0/2 | 1/2 | 1/2 |
|  |  |  |  |  |  |  |  |  |
|  | Snelling Rural | Jun. 5 | 37.51741, -120.34617 | 1 | *An. punctipennis* 6 | 0/1 | 0/1 | 0/1 |
|  |  |  |  |  |  |  |  |  |
|  | Snelling Rural | Jun. 12 | 37.51078, -120.34822 | 3 | An. punctipennis 7, 8, 9 | 0/3 | 0/3 | 0/3 |
|  |  |  |  |  |  |  |  |  |
|  | Snelling Rural | Jun. 12 | 37.51077, -120.34827 | 2 | An. punctipennis 10, 11 | 0/2 | 0/2 | 0/2 |
|  |  |  |  |  |  |  |  |  |
|  | Snelling Rural | Jun. 12 | 37.50989, -120.34576 | 2 | *An. punctipennis* 12, 13 | 0/2 | 0/2 | 0/2 |
|  |  |  |  |  |  |  |  |  |
|  | Snelling Rural | Jun. 21 | 37.51077, -120.34822 | 2 | *An. punctipennis* 14, 15 | 0/2 | 0/2 | 0/2 |
|  |  |  |  |  |  |  |  |  |
|  | Snelling Rural | Jun. 21 | 37.51078, -120.34828 | 1 | *An. punctipennis* 16 | 0/1 | 0/1 | 0/1 |
|  |  |  |  |  |  |  |  |  |
|  | Snelling Rural | Jun. 21 | 37.51734, -120.34626 | 1 | *An. punctipennis* 17 | 0/1 | 0/1 | 0/1 |
|  |  |  |  |  |  |  |  |  |
|  | Snelling Rural | Jul. 11 | 37.51078, -120.34820 | 1 | *An. punctipennis* 18 | 0/1 | 0/1 | 0/1 |
|  |  |  |  |  |  |  |  |  |
|  | Los Banos Rural | Sept. 18 | 37.19358, -120.26038 | 1 | *An. punctipennis* 19 | 0/1 | 0/1 | 0/1 |
|  |  |  |  |  |  |  |  |  |
|  |  |  |  |  | # screened per gene, combined: | 0/19 | 1/19 | 1/19 |
| *Culex pipiens* | 2018 |  |  |  |  |  |  |  |
|  | Merced Urban 2 | Jun. 7 | 37.312447, -120.465797 | 6 | *Cx. pipiens* 71-75 | 5/5 | 5/5 | 5/5 |
|  | Merced Urban 1 | Jun. 22 | 37.30802, -120.45249 | 6 | *Cx. pipiens* 66-70 | 5/5 | 5/5 | 5/5 |
|  | Atwater Rural 1 | Jun. 28 | 37.30318, -120.61743 | 6 | *Cx. pipiens* 76-80 | 5/5 | 5/5 | 5/5 |
|  | Ballico Rural | Jul. 2 | 37.49212, -120.59812 | 35 | *Cx. pipiens* 28-42* | 15/15 | 15/15 | 15/15 |
|  | Snelling Rural | Jul. 11 | 37.51078, -120.3482 | 2 | *Cx. pipiens* 21, 22 | 0/2 | 0/2 | 0/2 |
|  |  |  |  |  |  |  |  |  |
|  | Snelling Rural | Jul. 11 | 37.51079, -120.34826 | 1 | *Cx. pipiens* 23 | 0/1 | 0/1 | 0/1 |
|  |  |  |  |  |  |  |  |  |
|  | Snelling Rural | Jul. 11 | 37.51735, -120.34624 | 1 | *Cx. pipiens* 24 | 0/1 | 0/1 | 0/1 |
|  |  |  |  |  |  |  |  |  |
|  | Snelling Rural | Jul. 11 | 37.51739, -120.34617 | 3 | *Cx. pipiens* 25-27* | 1/3 | 1/3 | 1/3 |
|  |  |  |  |  | # screened per gene, combined: | 31/37 | 31/37 | 31/37 |
| *Culex stigmatosoma* | 2018 |  |  |  |  |  |  |  |
|  | Snelling Rural | Jun. 5  2018 | 37.51734, -120.34623 | 1 | *Cx. stig 35* | 0/1 | 0/1 | 0/1 |
|  |  |  |  |  |  |  |  |  |
|  | Snelling Rural | Jun. 21  2018 | 37.51078, -120.34828 | 1 | *Cx. stig* 36 | 0/1 | 0/1 | 0/1 |
|  |  |  |  |  |  |  |  |  |
|  | Merced Urban 1 | Jun. 22 | 37.30803, -120.45270 | 1 | *Cx. stig* 37 | 0/1 | 0/1 | 0/1 |
|  |  |  |  |  |  |  |  |  |
|  | Ballico Rural | Jun. 27 | 37.49213, -120.59809 | 3 | *Cx. stig* 17*-19 | 3/3 | 3/3 | 3/3 |
|  |  |  |  |  |  |  |  |  |
|  | Ballico Rural | Jun. 27 | 37.49215, -120.59813 | 5 | *Cx. stig* 20*-24 | 5/5 | 5/5 | 5/5 |
|  |  |  |  |  |  |  |  |  |
|  | Ballico Rural | Jun. 27 | 37.49248, -120.59816 | 5 | *Cx. stig* 25*-29 | 5/5 | 5/5 | 5/5 |
|  |  |  |  |  |  |  |  |  |
|  | Ballico Rural | Jun. 27 | 37.49245, -120.59838 | 2 | *Cx. stig* 30, 31 | 2/2 | 2/2 | 2/2 |
|  |  |  |  |  |  |  |  |  |
|  | Ballico Rural | Jun. 27 | 37.49276, -120.59855 | 1 | *Cx. stig* 32 | 1/1 | 1/1 | 1/1 |
|  |  |  |  |  |  |  |  |  |
|  | Ballico Rural | Jun. 27 | 37.49332, -120.59822 | 2 | *Cx. stig* 33, 34 | 2/2 | 2/2 | 2/2 |
|  |  |  |  |  |  |  |  |  |
|  | Atwater Rural 1 | Jun. 28 | 37.30318, -120.61743 | 1 | *Cx. stig* 38* | 1/1 | 1/1 | 1/1 |
|  |  |  |  |  |  |  |  |  |
|  | Atwater Rural 1 | Jun. 28 | 37.30338, -120.61756 | 1 | *Cx. stig* 39* | 1/1 | 1/1 | 1/1 |
|  |  |  |  |  |  |  |  |  |
|  | Ballico Rural | Jul. 2 | 37.49212, -120.59812 | 3 | *Cx. stig* 7*-9 | 3/3 | 3/3 | 3/3 |
|  |  |  |  |  |  |  |  |  |
|  | Ballico Rural | Jul. 2 | 37.49218, -120.59813 | 3 | *Cx. stig* 10*-12 | 3/3 | 3/3 | 3/3 |
|  |  |  |  |  |  |  |  |  |
|  | Ballico Rural | Jul. 2 | 37.49243, -120.59838 | 2 | *Cx. stig* 13*, 14 | 2/2 | 2/2 | 2/2 |
|  |  |  |  |  |  |  |  |  |
|  | Ballico Rural | Jul. 2 | 37.49274, -120.59850 | 1 | *Cx. stig* 15* | 1/1 | 1/1 | 1/1 |
|  |  |  |  |  |  |  |  |  |
|  | Ballico Rural | Jul. 2 | 37.49331, -120.59822 | 1 | *Cx. stig* 16* | 1/1 | 1/1 | 1/1 |
|  |  |  |  |  |  |  |  |  |
|  | Snelling Rural | Aug. 20 | 37.51077, -120.34822 | 1 | *Cx. stig* 40 | 0/1 | 0/1 | 0/1 |
|  |  |  |  |  |  |  |  |  |
|  |  |  |  |  | # screened per gene, combined: | 30/34 | 30/34 | 30/34 |
| *Culex tarsalis* | 2018 |  |  |  |  |  |  |  |
|  | Le Grand Rural | Jun. 19 | 37.19358, -120.26038 | 4 | *Cx. tarsalis* 57-60 | 0/4 | 0/4 | 0/4 |
|  |  |  |  |  |  |  |  |  |
|  | Le Grand Rural | Jun. 19 | 37.19351, -120.26042 | 6 | *Cx. tarsalis* 61-66 | 0/1 | 0/1 | 0/1 |
|  |  |  |  |  |  |  |  |  |
|  | Merced Rural 1 | Jun. 28 | 37.31892, -120.42629 | 12 | *Cx. tarsalis* 77-80 | 0/4 | 0/4 | 0/4 |
|  |  |  |  |  |  |  |  |  |
|  | Ballico Rural | Jul. 2 | 37.49243, -120.59838 | 11 | *Cx. tarsalis* 40-50 | 0/11 | 1/11 | 1/11 |
|  |  |  |  |  |  |  |  |  |
|  | Ballico Rural | Jul. 2 | 37.49274, -120.59850 | 1 | *Cx. tarsalis* 51 | 0/1 | 0/1 | 0/1 |
|  |  |  |  |  |  |  |  |  |
|  | Ballico Rural | Jul. 2 | 37.49331, -120.59822 | 3 | *Cx. tarsalis* 52-54 | 0/3 | 0/3 | 0/3 |
|  |  |  |  |  |  |  |  |  |
|  | Snelling Rural | Jul. 11 | 37.51078, -120.34820 | 3 | *Cx. tarsalis* 55, 56 | 0/2 | 0/2 | 0/2 |
|  |  |  |  |  | # screened per gene, combined: | 0/26 | 1/26 | 1/26 |
| *Culiseta incidens* | 2017 |  |  |  |  |  |  |  |
|  | Merced Urban Site 37 | 9/19/17 | 37.31273, -120.48699 | 20 | *Cs. incidens* 75-77; 78-90 | 0/16 | 0/16 | 0/16 |
|  |  |  |  |  |  |  |  |  |
|  | Merced Urban Site 37 | 10/3/17 | 37.31273, -120.48699 | 9 | *Cs. incidens* 66-74 | 0/9 | 0/9 | 0/9 |
|  |  |  |  |  |  |  |  |  |
|  | Merced Urban Site 37 | 10/4/17 | 37.31273, -120.48699 | 2 | *Cs. incidens* 64, 65 | 0/2 | 0/2 | 0/2 |
|  |  |  |  |  |  |  |  |  |
| *Culiseta incidens* | 2018 |  |  |  |  |  |  |  |
|  | Snelling Rural | Jun. 5  2018 | 37.51080, -120.34821 | 1 | *Cs. incidens* 47 | 0/1 | 0/1 | 0/1 |
|  |  |  |  |  |  |  |  |  |
|  | Snelling Rural | Jun. 5 | 37.509970, -120.34576 | 1 | *Cs. incidens* 48 | 0/1 | 0/1 | 0/1 |
|  |  |  |  |  |  |  |  |  |
|  | Snelling Rural | Jun. 5 | 37.51741, -120.34617 | 1 | *Cs. incidens* 49 | 0/1 | 0/1 | 0/1 |
|  |  |  |  |  |  |  |  |  |
|  | Merced Urban 2 | Jun. 7 | 37.3126, -120.4658 | 2 | *Cs. incidens* 50, 51 | 0/2 | 0/2 | 0/2 |
|  |  |  |  |  |  |  |  |  |
|  | Merced Urban 2 | Jun. 7 | 37.3126, -120.4658 | 1 | *Cs. incidens* 52 | 0/1 | 0/1 | 0/1 |
|  |  |  |  |  |  |  |  |  |
|  | Le Grand Rural | Jun. 19 | 37.19405, -120.26173 | 1 | *Cs. incidens* 53 | 0/1 | 0/1 | 0/1 |
|  |  |  |  |  |  |  |  |  |
|  | Snelling Rural | Jun. 21 | 37.51078, -120.34828 | 1 | *Cs. incidens* 54 | 0/1 | 0/1 | 0/1 |
|  |  |  |  |  |  |  |  |  |
|  | Snelling Rural | Jun. 21 | 37.51734, -120.34626 | 1 | *Cs. incidens* 55 | 0/1 | 0/1 | 0/1 |
|  |  |  |  |  |  |  |  |  |
|  | Snelling Rural | Jun. 21 | 37.51738, -120.34620 | 1 | *Cs. incidens* 56 | 0/1 | 0/1 | 0/1 |
|  |  |  |  |  |  |  |  |  |
|  | Merced Urban 1 | Jun. 22 | 37.30803, -120.45270 | 2 | *Cs. incidens* 57, 58 | 0/2 | 0/2 | 0/2 |
|  |  |  |  |  |  |  |  |  |
|  | Merced Urban 1 | Jun. 22 | 37.30803, -120.45270 | 4 | *Cs. incidens* 59-60 | 1/2 | 1/2 | 1/2 |
|  |  |  |  |  |  |  |  |  |
|  | Merced Urban 1 | Jun. 22 | 37.30803, -120.45270 | 1 | *Cs. incidens* 63 | 0/1 | 0/1 | 0/1 |
|  |  |  |  |  |  |  |  |  |
|  |  |  |  |  | # screened per gene, combined: | 1/42 | 1/42 | 1/42 |
| *Culiseta inornata* | 2017 |  |  |  |  |  |  |  |
|  | Snelling Rural Site 2 | 7/12/17 | 37.51076, -120.34823 | 1 | *Cs. inornata* 1* | 0/1* | 1/1 | 1/1 |
|  |  |  |  |  |  |  |  |  |
|  | Merced Urban Site 2 | 8/7/17 | 37.35344, -120.47296 | 1 | *Cs. inornata* 2 | 0/1 | 0/1 | 0/1 |
|  |  |  |  |  |  |  |  |  |
| *Culiseta inornata* | 2019* |  |  |  |  |  |  |  |
|  | Snelling Rural Site 1 | 11/13/19 | 37.51078, -120.3482 | 2 | *Cs. inornata* 3,4 | 0/2 | 0/2 | 0/2 |
|  |  |  |  |  |  |  |  |  |
|  | Snelling Rural Site 2 | 11/13/19 | 37.51078, -120.3482 | 2 | *Cs. inornata* 5,6 | 0/2 | 0/2 | 0/2 |
|  |  |  |  |  |  |  |  |  |
|  | Snelling Rural Site 5 | 11/13/19 | 37.51735, -120.34624 | 1 | *Cs. inornata* 7 | 0/1 | 0/1 | 0/1 |
|  |  |  |  |  |  |  |  |  |
|  |  |  |  |  | # screened per gene, combined: | 1/7 | 1/7 | 1/7 |

**Supplemental Table S1.** Mosquito collections in Merced county. Samples designated with an asterisk have been selected for *Wolbachia* Supergroup characterization by MLST. Samples include *Culex pipiens* 27-36, *Culex stigmatosoma* 7, 10, 13, 15, 16, 17, 20, 25, 38, 39, and *Culiseta inornata* 1. *Culiseta inornata* 1 and 2 were only tested with 16srRNA
